# Solid Fuel Use for Household Cooking: Country and Regional Estimates for 1980–2010

**DOI:** 10.1289/ehp.1205987

**Published:** 2013-05-03

**Authors:** Sophie Bonjour, Heather Adair-Rohani, Jennyfer Wolf, Nigel G. Bruce, Sumi Mehta, Annette Prüss-Ustün, Maureen Lahiff, Eva A. Rehfuess, Vinod Mishra, Kirk R. Smith

**Affiliations:** 1 Department of Public Health and Environment, World Health Organization, Geneva, Switzerland; 2 Department of Environmental Health Sciences, School of Public Health, University of California, Berkeley, Berkeley, California, USA; 3 Department of Public Health and Policy, Institute of Psychology, Health and Society, University of Liverpool, Liverpool, United Kingdom; 4 Global Alliance for Clean Cookstoves, Washington, DC, USA; 5 Division of Epidemiology, School of Public Health, University of California, Berkeley, Berkeley, California, USA; 6 Institute for Medical Informatics, Biometry and Epidemiology, Ludwig-Maximilians-Universität Munich, Munich, Germany; 7 Population Division, Department of Economic and Social Affairs, United Nations, New York, New York, USA

**Keywords:** biomass fuel, coal, cookstoves, disease burden, household air pollution, household energy, indoor air pollution, MDGs, Millennium Development Goals

## Abstract

Background: Exposure to household air pollution from cooking with solid fuels in simple stoves is a major health risk. Modeling reliable estimates of solid fuel use is needed for monitoring trends and informing policy.

Objectives: In order to revise the disease burden attributed to household air pollution for the Global Burden of Disease 2010 project and for international reporting purposes, we estimated annual trends in the world population using solid fuels.

Methods: We developed a multilevel model based on national survey data on primary cooking fuel.

Results: The proportion of households relying mainly on solid fuels for cooking has decreased from 62% (95% CI: 58, 66%) to 41% (95% CI: 37, 44%) between 1980 and 2010. Yet because of population growth, the actual number of persons exposed has remained stable at around 2.8 billion during three decades. Solid fuel use is most prevalent in Africa and Southeast Asia where > 60% of households cook with solid fuels. In other regions, primary solid fuel use ranges from 46% in the Western Pacific, to 35% in the Eastern Mediterranean and < 20% in the Americas and Europe.

Conclusion: Multilevel modeling is a suitable technique for deriving reliable solid-fuel use estimates. Worldwide, the proportion of households cooking mainly with solid fuels is decreasing. The absolute number of persons using solid fuels, however, has remained steady globally and is increasing in some regions. Surveys require enhancement to better capture the health implications of new technologies and multiple fuel use.

Cooking with solid fuels (biomass such as wood, crop residues, dung, charcoal, and coal) over open fires or in simple stoves exposes household members to daily pollutant concentrations that lie between those of second-hand smoke and active smoking (Pope et al. 2009, 2011; Smith and Peel 2010). Based on the results of the first comparative risk assessment (CRA) of the Global Burden of Disease (GBD) project (Smith et al. 2004), this practice was estimated to cause about 2 million premature deaths from pneumonia, chronic obstructive pulmonary disease, and lung cancer [World Health Organization (WHO) 2009]. The GBD 2010 project, published in 2012, which used the household fuel estimates reported here, found household air pollution to be responsible for 3.5 million premature deaths globally, and includes other health outcomes, such as cataracts and cardiovascular diseases (Lim et al. 2012). In the GBD 2010, household cooking fuels also contributed substantially to outdoor air pollution in many regions and, as a result, was responsible for about half a million more premature deaths (Lim et al. 2012). Additional impacts of household solid fuel use on health, not currently included in disease burden estimates, derive from adverse pregnancy outcomes (Pope et al. 2010), the risk of burns and scalds, and the risk of injury and violence during fuel collection (WHO 2006), as well as the contribution to ambient (outdoor) air pollution. Many solid-fuel users are forced to spend a significant amount of time gathering fuel, time which otherwise could be used for income-generating or child care activities or schooling (WHO 2006). Furthermore, inefficient use of solid fuels in households has important impacts on the local environment as well as global climate change (Edwards et al. 2004; Smith et al. 2000).

In view of the public health, social, and environmental impacts of household solid fuel use, capturing the current rate and trends is critical to inform policy across various sectors (e.g., energy, environment, health). The indicator “solid fuel use” (SFU) serves as input for the estimation of health impacts in the GBD 2010’s CRA [Institute for Health Metrics and Evaluation (IHME) 2012]. SFU is reported in the World Health Statistics series (WHO 2012a) and, until 2007, was also a Millennium Development Goal (MDG) indicator for environmental sustainability (United Nations 2007, 2012a).

Estimates of the proportion of households in a country using solid fuels as their main energy source for cooking are reported in household surveys, but the estimates need to be modeled for the purpose of monitoring trends and providing point estimates for countries and regions in specific years. In the past, with relatively few nationally representative household fuel surveys to use, estimates relied simply on the latest available survey point or used linear regression analysis, with or without covariates at the national level (Mehta et al. 2006; Rehfuess et al. 2006; Smith et al. 2004; WHO 2010). In recent years, the number of available surveys has increased substantially, allowing for more empirical modeling based closely on available data points.

In the present study, we first estimated annual household SFU for cooking over the 30-year period 1980 to 2010, by country and region, based on transparent and reproducible methods that rely heavily on available national survey information. The outputs met data requirements for the CRA/GBD 2010 (IHME 2012) and for ongoing WHO reporting in the World Health Statistics series (WHO 2012a). We then evaluated trends in usage by region and certain specific countries, explored limitations in the database, and made suggestions for improving methods for data collection and reporting.

## Methods

*Data*. We used the data from the WHO household energy database (WHO 2012b), which is a systematic compilation of nationally representative surveys or censuses and builds on earlier versions developed by the University of California, Berkeley (Smith et al. 2004). The WHO database provides estimates of the percentage of households using as their primary cooking fuel solid fuels (coal, wood, charcoal, dung, and crop residues), liquid fuels (kerosene), gaseous fuels (liquid petroleum gas, natural gas, biogas), and electricity. About three-fourths of the data were disaggregated by individual fuel type and approximately two-thirds of the data by urban and rural residency. These estimates do not directly include fuels used for space heating.

These survey data were obtained from a variety of sources. International multicountry surveys, specifically Macro International’s Demographic and Health Surveys (U.S. Agency of International Development 2012), UNICEF’s Multiple Indicator Cluster Surveys (UNICEF 2012), the WHO’s World Health Surveys (WHO 2012c), and the World Bank’s Living Standard Measurement Studies (World Bank 2012b), which together account for 39% of data points in the database. National censuses constitute a further 18%, and other national surveys such as household, employment, living conditions, or expenditure surveys accounted for another 20% of the database. The remaining 23% of data points are from other sources, including environmental and poverty assessments, MDG reports, and statistical figures provided on the websites of national statistics bureaus.

A total of 586 national country-year data points were available for modeling. These data points covered 155 countries, including 97% of all low- and middle-income countries (LMIC; defined as having < US$12,276 per capita in 2011–2012) and territories between 1974 and 2010, with at least one survey per country. Further details are available in Supplemental Material, Table S1 (http://dx.doi.org/10.1289/ehp.1205987).

*Methods for modeling household SFU at the national level.* The aim of the modeling was to obtain a complete set of annual trends of primary SFU by country using a transparent, reproducible model. The model should be suitable for estimating SFU for years without survey information in a particular country, and for countries without any survey data. The model should also closely follow empirical data without being unduly influenced by large fluctuations in survey estimates of SFU over adjacent countries or years. This is important because large fluctuations are unlikely in practice and generally reflect (in addition to random error) differences in survey design and conduct. In the absence of data for certain periods, we borrowed information from regional trends, assuming that fuel use patterns are likely to be similar. Also, the model should not be unduly sensitive to parameters such as following the trends of covariates (e.g., gross national income per capita) without compelling evidence of similar trends in SFU.

As seen in other work estimating household SFU (Mehta et al. 2006), for countries with no solid-fuel data but that are classified as high-income countries according to the World Bank country classification (World Bank 2012a), SFU was assumed to be < 5%.

We reviewed range of alternative modeling approaches, including a variety of linear regression models and Bayesian hierarchical/Gaussian process regression models [for details see Supplemental Material, pp. 2–3 (http://dx.doi.org/10.1289/ehp.1205987)]. Also, potential developmental and energy-related covariates thought to be related to household solid fuel use (e.g., gross national income per capita, the percentage of the total population living in rural areas, population density, the percentage of the total population with access to improved sanitation, and the percentage of total energy consumption from fossil fuels) were investigated.

Multilevel/mixed-effects model. A multilevel nonparametric model without covariates was selected because it best fulfilled the above criteria and provided the best fit to the data based on Akaike’s information criterion (AIC), the Bayesian information criterion (BIC), and visual inspection. Modeling assumptions—linearity, normality, and homoscedasticity—also were checked by visual inspection of the residuals and were reasonably met (Goldstein 2010; Hox 2010). All surveys were included in the model [see Supplemental Material, Table S1 (http://dx.doi.org/10.1289/ehp.1205987)]. Covariates (income, percentage of rural population, population density) were evaluated but not retained because trends in some countries were rather sensitive to the particular set of covariates used. Multilevel modeling takes into account the hierarchical structure of the data; for example, survey points are correlated within countries, which are then clustered within regions (Goldstein 2010). When information is scarce for a particular country, regional information is used to derive estimates for a country.

The 155 countries were grouped into the 21 GBD regions, which are based on geographical proximity and epidemiological similarity (IHME et al. 2009). The model included hierarchical random effects for regions and countries. Time was the only explanatory variable included in the model, both in terms of fixed and random effects (at country level). The time variable was centered at the year 2003 (the median date of the surveys) and transformed into a natural cubic spline to allow for nonlinearity while providing a desired degree of stability (Orsini and Greenland 2011; Peng et al. 2006). The number of knots for the spline was chosen to allow the model to adequately follow the survey point trend and avoid any unlikely fluctuation. The locations of the knots were determined by the percentiles of the independent variable (Harrell 2001). The covariance model was chosen to be unstructured.

Using a technique of statistical simulation described by King et al. (2000), we computed the national SFU prevalence estimates and accounted for uncertainty. We drew 1,000 times from the model parameters for the fixed effects to generate the outcome variable in order to capture the estimation uncertainty. We used the method described by De Onis et al. (2004) to derive regional and global prevalence confidence intervals (CIs).

We used the multilevel model for 150 countries with at least one survey data point. Regional estimates were used instead of model estimates for seven LMICs without survey data. We tested this assumption by performing out-of-sample evaluations on a truncated data set by removing countries from the data set (repeated 30 times). The mean median percentage point difference between the withheld data and the regional mean was 15.8%. We performed additional out-of-sample evaluations on three truncated data sets *a*) with 20% of the country-years withheld on countries with more than one survey (repeated 30 times), *b*) with the last survey withheld in countries with more than one survey and, *c*) with the last 3 years (2008–2010) withheld. The median percentage point differences between the withheld data and the model outputs were 3.7%, 3.6%, and 3.7%, respectively.

Calculation of the population exposed. The model derives estimates of the percentage of households using solid fuels for a particular country and year. The fraction of the population exposed was assumed to be the same as the fraction of households using solid fuels. Accordingly, the SFU fraction was multiplied by the national population (United Nations 2012b) to obtain an estimate of the absolute population exposed per country. In other words, no attempt was made to adjust population estimates for variations in household size across various settings (e.g., urban vs. rural households) because such data were not consistently available.

All analyses were conducted using Stata software (version 12; StataCorp LP, College Station, TX, USA).

## Results

A complete data series of households mainly using solid fuels for cooking was generated for 150 countries using hierarchical modeling from 1980 to 2010 [see Supplemental Material, Tables S2–S4 (http://dx.doi.org/10.1289/ehp.1205987)]. The national surveys used in deriving this model represented 85% of the 2010 world population. For the seven countries without any survey information [i.e., Bulgaria, Equatorial Guinea, Hungary, Kiribati, Lithuania, Poland, and the Federation of Saint Kitts and Nevis (1% of world population)] regional estimates were used. A total of 36 wealthier (> US$12,276 per capita) countries without survey data were assumed to have made the transition to clean fuels with < 5% SFU, accounting for the remaining 14% of the world population.

**Figure 1:**
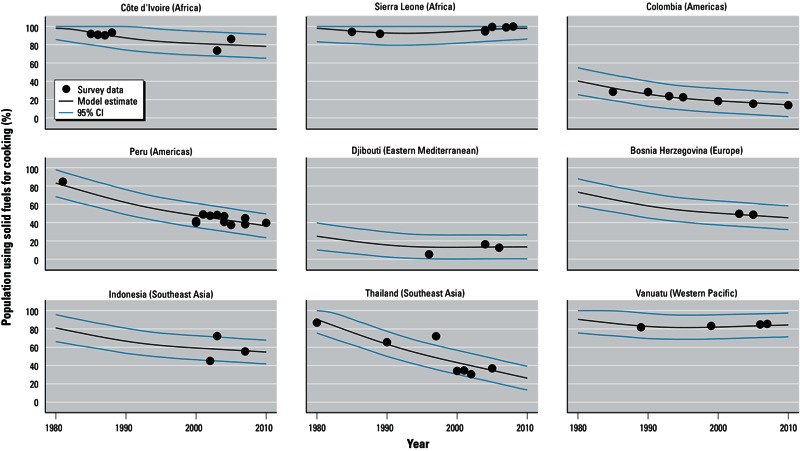
Trends of population using solid fuels as main cooking fuel in selected LMICs (low- and middle-income countries): model results compared to actual survey data, 1980–2010. Countries are grouped by WHO region and income category [WHO 2012e; see Supplemental Material, Table S2 (http://dx.doi.org/10.1289/ehp.1205987)].

Figure 1 provides an example of the modeled trends for at least one country per region, and examples of countries with many or few survey data points, or with survey data spread out or clustered over time. It demonstrates the hierarchical model’s ability to provide annual estimates at or near the survey points reported by households surveys while also following regional trends in the absence of survey points. Even in these few examples, the marked differences in country trends are highlighted, including steep declines (e.g., Thailand, Peru), relatively stable patterns (e.g., Côte d’Ivoire, Djibouti), and increases in SFU (Sierra Leone and Vanuatu).

**Figure 2:**
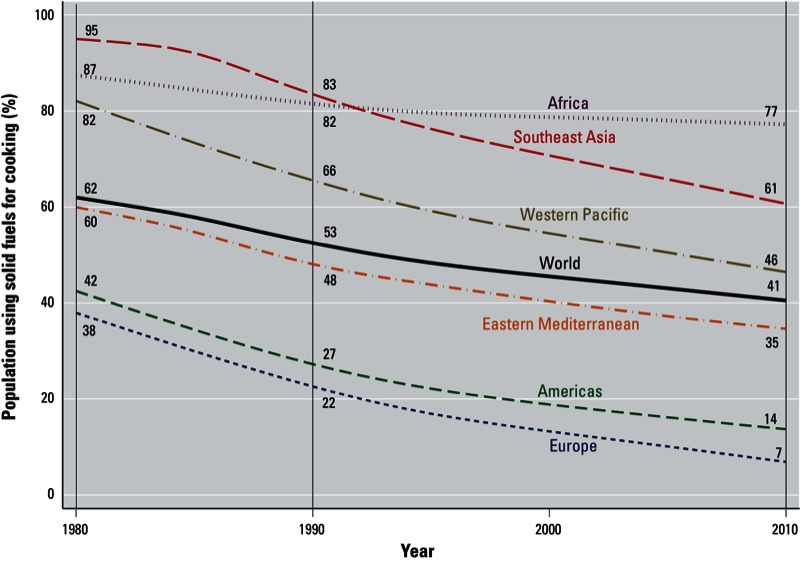
Regional trends for the percentage of population using solid fuels as the main cooking fuel in LMICs (low- and middle-income countries), 1980–2010. Countries are grouped by WHO region and income category (WHO 2012e; see Supplemental Material, Table S2 (http://dx.doi.org/10.1289/ehp.1205987). 95% CIs for the years 1990, 2000, and 2010 are given in Supplemental Material, Table S3.

The proportion of the world’s households primarily relying on solid fuels for cooking declined from 62% (95% CI: 58, 66) to 41% (95% CI: 37, 44) between 1980 and 2010 [Figure 2 and see also Supplemental Material, Table S3 (http://dx.doi.org/10.1289/ehp.1205987)]. Proportions have steadily decreased for all regions since 1980, and only in Sub-Saharan Africa (hereafter referred to as Africa, North Africa being part of the Eastern Mediterranean region) was the decline notably slower. Africa and Southeast Asia are the regions with the highest proportion of households using solid fuels with 77% (95% CI: 74, 81) and 61% (95% CI: 52, 70), respectively, in 2010; whereas Europe and the Americas are the lowest, with < 20%. The Western Pacific and Eastern Mediterranean regions lie in the mid-range, with 46% (95% CI: 35, 57) and 35% (95% CI: 29, 40), respectively. In high-income countries, solid fuels are used by < 5% of the population (not shown). The decline has been sharpest in Asia (both Western Pacific and Southeast Asia).

**Figure 3:**
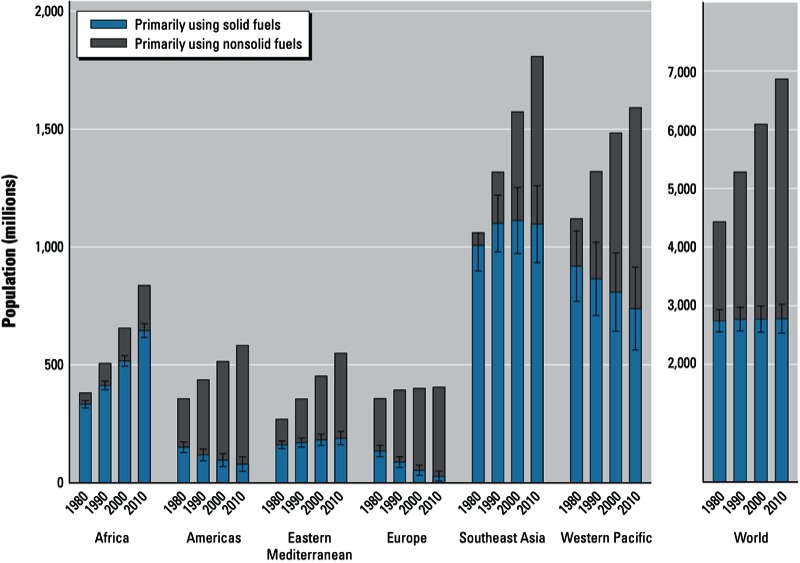
Global and regional trends ± 95% CIs in population relying on solid fuels as the main cooking fuel in LMICs (low- and middle-income countries), from 1980 to 2010. Countries are grouped by WHO region and income category (WHO 2012e; see Supplemental Material, Table S2 (http://dx.doi.org/10.1289/ehp.1205987). 95% CIs for the years 1990, 2000, and 2010 are given in Supplemental Material, Table S4.

Despite declines in the proportions of households using solid fuels for cooking, the absolute number of persons mainly using solid fuel for cooking has remained stable over the last three decades—at around 2.7 billion to 2.8 billion—due to population growth [Figure 3; see also Supplemental Material, Table S4 (http://dx.doi.org/10.1289/ehp.1205987)]. Unlike in other regions, the number of households using solid fuels almost doubled in Africa, from 333 million to 646 million, and slightly increased in the Eastern Mediterranean region, from 162 million to 190 million. In Southeast Asia, the number has remained stable in terms of households exposed, whereas it declined in Europe, the Americas, and the Western Pacific.

**Figure 4:**
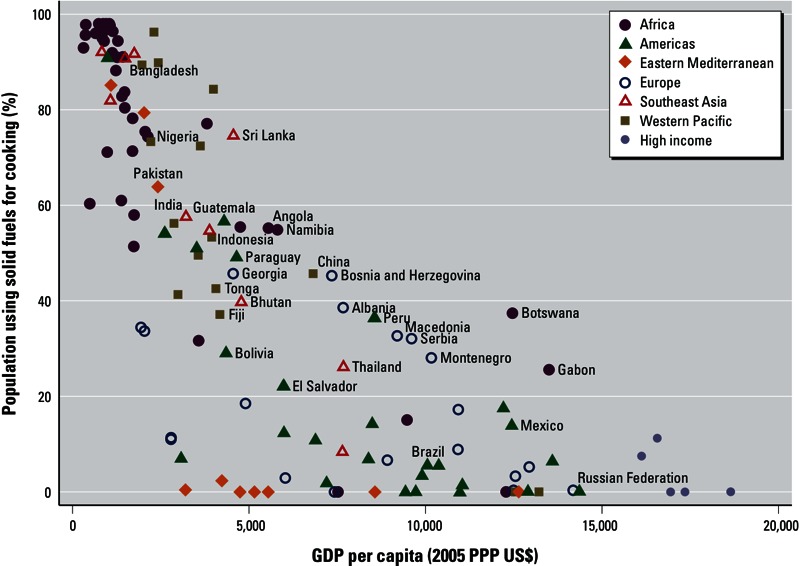
Percentage of population using solid fuels as main cooking fuel versus gross domestic product (GDP) per capita, 2010 [adjusted by 2005 US$ purchasing power parity (PPP)]. Source for GDP: World Bank (Azevedo 2011). Country names are displayed for selected countries; countries are color-coded by WHO region and income category [WHO 2012e; see Supplemental Material, Table S2 (http://dx.doi.org/10.1289/ehp.1205987)].

Figure 4 presents estimated SFU prevalences for 2010 by country in relation to income level. SFU remains closely associated with national income; however, it is apparent that for the same national income level, household solid fuel use for cooking can vary considerably. Factors in this variation include differences in the availability of biomass, coal, and alternative cleaner fuels; the distribution of income within the country; and the degree of urbanization.

**Figure 5:**
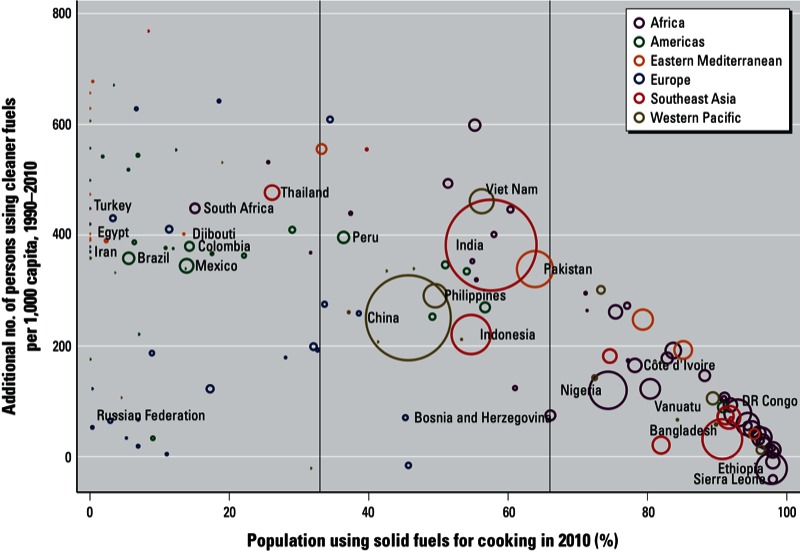
Progress in using cleaner fuels as main cooking fuel between 1990 and 2010, by country. The size of each circle is proportional to the absolute number of households using solid fuels as main fuel for cooking. Country names are displayed for selected countries; countries are color-coded by WHO region and income category [WHO 2012e; see Supplemental Material, Table S2 (http://dx.doi.org/10.1289/ehp.1205987)].

Progress since 1990 in terms of number of persons using cleaner fuels as their main cooking fuel is shown in Figure 5. Only 60 countries—of which 47 are LMIC—have reduced the portion of the population without access to modern cooking fuels by 50% (which corresponds to the formulation of the previous MDG), and these are mainly countries that previously had limited use of solid fuels (< 33%). In Figure 5, three groups can be distinguished:

Countries with less than one third of their population using solid fuels: These countries have relatively small populations using solid fuels as their main cooking fuel, and although their progress has been mixed, they have at least halved their populations exposed to household solid-fuel combustion between 1990 and 2010.
Countries with between one-third and two-thirds of their population relying on solid fuels as their main cooking fuel: Countries with a large percentage of the population using solid fuels in this group have made important progress between 1990 and 2010 (i.e., an additional 200–500 persons/1,000 inhabitants now use cleaner fuels as their main cooking fuel); these countries include China, India, Indonesia, and Pakistan.
Countries with more than two-thirds of their population using solid fuels as their main fuel for cooking: Overall these countries appear to have made more limited progress and are mainly clustered within Africa.

## Discussion

Compared with previous assessments, the SFU estimates presented here are slightly lower: Although global SFU prevalence was assessed to be 57% for the year 2000 (Smith et al. 2004), 52% for the year 2003 (Rehfuess et al. 2006), and 42% for 2007 (WHO 2010), our model predicts 53% for 1990, 43% for 2005, and 41% for 2010. The differences can be explained by both the methodologies used to derive the estimates and the greater number of surveys used to develop the model (534 additional surveys have become available since 2000). Currently, 139 of the 144 LMIC are covered by at least one survey, whereas in the year 2000, 92 had no survey information.

Our multilevel model closely follows the empirical data without responding unduly to fluctuations, in a transparent and reproducible way. For countries with several survey data points, the model was able to provide estimates close or equal to the empirical data. For countries with few data points, information borrowed from the regions provided likely trends, with the underlying assumptions that *a*) regional trends are better predictors than the available information at national level, and *b*) countries within the same region are similar in terms of energy access and cultural habits.

Estimating the proportion of the population relying mainly on solid fuel use for cooking is important because of its links to smoke exposure and the associated health impacts. Thus, primary SFU has been the main indicator successfully used in epidemiological studies for a range of diseases in children and adults to determine the risks of exposure to air pollution in the household environment. However, the proportion of the population exposed is likely estimated because, in most countries, use of solid fuels for cooking is more common among poorer households, which tend to have higher fertility and larger family size than those using cleaner, nonsolid fuels (Gwatkin et al. 2007).

Although biomass fuels contain few actual contaminants and are not intrinsically dirty, they produce substantial pollution mainly as a result of incomplete combustion in traditional stoves and open fires. Unfortunately, in developing countries today, few truly advanced combustion biomass cookstoves that reduce the emission levels in the household environment to levels safe for health are in use (WHO 2012d). Cooking with biomass in developing countries is therefore essentially equivalent to harmful exposure.

However, as advanced stoves come into more widespread use over time, SFU by itself will become increasingly problematic as an indicator for impacts from household combustion in terms of health, climate, and environment. Survey questions will need to capture the difference between “clean” and “dirty” SFU by combining this information with the type and condition of combustion device being used (e.g., cookstove) as well as any secondary or tertiary fuel and technology sources and stove ventilation (e.g., smoke hoods, chimneys).

This risk factor has previously been defined as “indoor air pollution from solid fuel use.” More recently, however, the field has adopted the term “household air pollution” as a more accurate term to describe its health and environmental impacts. This is because health-damaging exposures from cookstoves occur not only in the kitchen but in and around the home; likewise, health effects are similarly observed among populations that predominantly cook outdoors. Air monitoring studies have shown that in many homes using solid fuels, the smoke produced during cooking activities leaks into other rooms and areas directly surrounding the home, where household members spend a lot of their time (Balakrishnan et al. 2004). These pollutant levels, although often occurring at lower levels, can still be health damaging (Smith et al. 2010).

In addition, in communities where solid fuel is commonly used, households that rely mainly on clean fuel and/or advanced combustion technologies may still be chronically exposed to high levels of air pollution caused by smoke-producing neighboring households, that is, “neighborhood pollution” (Naeher et al. 2000). This implies that solutions should focus on reducing emissions through the use of cleaner fuels and technologies, rather than simply routing smoke outdoors through chimneys or smoke hoods, and should address whole communities rather than single households.

Another understanding that has only been quantified in recent years is that introduction of new cooking fuels and stoves in many areas is best described as a “stacking” process: New devices do not usually substitute 100% for old ones, but rather are initially used for certain cooking tasks and over time, perhaps slowly, displace older devices across most or all household energy tasks (Ruiz-Mercado et al. 2011). For an improved assessment of exposure, surveys should assess information on all fuels and devices used for cooking and other end uses (i.e., heating, lighting), rather than on the main fuel for cooking alone.

Space heating and other applications (such as uses of solid fuels for heating of water, use of incense, or “recreational” solid fuel use in fireplaces,) were not included in this analysis because they are not routinely reported in surveys and they involve different interventions. However, these uses can also result in harmful exposures and substantially contribute to ambient air pollution (Lei et al. 2011; Smith and Pillarisetti 2012; Ward and Lange 2010). An examination of this issue is warranted but is not included here.

Although we have included coal as a solid fuel in this analysis, its health and environmental impacts depart significantly from biomass. Unlike biomass, coal contains intrinsic contaminants, commonly including sulfur, mercury, and ash, but also, depending on the quality of the coal, arsenic, fluorine, lead, and other toxic constituents. This makes it difficult to generalize, although, like biomass, if burned in unprocessed forms, coal will also produce significant pollution in the form of products of incomplete combustion (International Agency for Research on Cancer 2010).

Another limitation of SFU as a framing of health risk is that processed solid fuels have different combustion characteristics than unprocessed solid fuels. For example, biomass pellets are much easier to burn cleanly than unprocessed wood or crop residues. As their use increases, this should be accounted for in surveys and other data collection efforts. Even processed coal, although difficult to truly burn cleanly, can be made safer through the removal of contaminants. Finally, charcoal, which is commonly used for cooking in Africa and the Caribbean, is included here as a solid fuel. Although charcoal produces fewer particulate emissions in simple stoves than does wood, it poses other risks to health from the pollution released during its manufacture in simple kilns, and from the high concentrations of carbon monoxide released during its use, in some cases leading to overnight poisonings in households (Smith 1987).

Finally, this modeling exercise did not consider kerosene, which is used by a significant fraction of LMIC populations for cooking (data not shown). In addition, many low-income households rely heavily on lighting with kerosene wick lamps, which are increasingly recognized as an important contributor to household air pollution and disease (Pokhrel et al. 2010). Due to increasing concerns about the health impacts of kerosene, this fuel should not be considered clean. Thus, although SFU can be used as a reasonable indicator of pollution exposures, the inverse, non-SFU, should not be considered an indicator of clean fuel use in populations relying on kerosene (Lam et al. 2012).

As discussed above, the use of primary cooking fuel as an indicator of exposure to household air pollution is not perfect, but it still is the single best global indicator that can be derived from population-based sources using a relatively standardized methodology. Cooking practices—including type of stove and cooking duration, division of tasks among household members, and location of the kitchen—may have an important impact on the actual personal exposure (Ruiz-Mercado et al. 2011) that may not be reflected by the use of solid fuels for cooking alone.

## Conclusion

A reliable and empirically based model was developed to generate annual national estimates of solid fuel use over 30 years for the GBD 2010 project and for international reporting purposes. The nonparametric multilevel model was used to estimate the percentage of households relying on solid fuel for 150 countries.

Although the proportion of households using solid fuels as their primary cooking fuel decreased in all regions from 1980 to 2010 to reach 41% globally, the actual number of persons exposed to household air pollution resulting from the use of these traditional fuels has remained stable at roughly 2.8 billion. By WHO region, there are three major trends:

In Africa, the number of SFU households is increasing, with 77% prevalence in 2010 and 646 million persons exposed. The Eastern Mediterranean region experienced a slight increase in the population exposed although prevalence has fallen.
In Southeast Asia, there has been a substantial decrease in the percentage of SFU from 95% to 61%, but population growth has kept the population cooking with solid fuels at around 1 billion.
Europe, the Americas, and the Western Pacific regions have experienced declines in both SFU prevalence and the populations exposed.
A more comprehensive assessment of energy use within the home would provide a better understanding of the health and environmental impacts of household air pollution.

Some suggestions for improving the quality and utility of household energy data for research and policy planning purposes include the following:

Collection and reporting of fuels and technologies used for other household energy uses (i.e., heating, lighting)
Collection and reporting of technologies (i.e., type of stove) as adoption of more advanced combustion devices increases
Collection and reporting of secondary fuels and technologies used for all end uses within the home
Better disaggregation of data by individual fuel type, including reporting data for processed and contaminated fuels.

Exposure to household air pollution is estimated to be among the most important causes of ill health in poor countries; using the estimates reported here for solid fuel use, the GBD 2010 found household air pollution to be the second most important risk factor for women and girls globally and fourth overall among those examined. In many poor countries, it is ranked first (Lim et al 2012). Despite this knowledge, the number of persons exposed to household air pollution has remained essentially unchanged. Additional insights into effective solutions to reduce exposure to household air pollution, allied with the currently high profile of energy issues on the global agenda at the moment [i.e., Global Alliance for Clean Cookstoves 2010 (http://www.cleancookstoves.org); UN Secretary General’s Year of Sustainable Energy for All, Rio+ 20 UN Conference on Sustainable Development] should result in increased access to clean cookstoves and fuels, as well as other innovative solutions to reduce health risks worldwide.

In common with many poverty-related indicators, the historical trend of household solid fuel use presents a mixed story. More persons are gaining access to modern, clean fuels over time. Importantly, because of population growth, however, the absolute impact is not declining. Indeed, there are more persons using such fuels today than anytime in human history. It is these absolute numbers that tell the story of potential impacts, both on households and on the global environment.

## Supplemental Material

(770 KB) PDF
