# Supplementary material for: Solid Fuel Use for Household Cooking: Country and Regional Estimates for 1980–2010
Source: Environ Health Perspect. 2013 May 3;121(7):784–90. doi: 10.1289/ehp.1205987 (PMC3701999; doi:10.1289/ehp.1205987)
Supplement: (770 KB) PDF [file ehp.1205987.s001.pdf]

Supplemental Material
Solid Fuel Use for Household Cooking: Country and Regional Estimates for 1980-2010

Sophie Bonjour, Heather Adair-Rohani, Jennyfer Wolf, Nigel G. Bruce, Sumi Mehta, Annette Prüss-Üstün, Maureen Lahiff, Eva A. Rehfuess, Vinod Mishra, Kirk R. Smith

Contents

Supplemental Material, Table S1. Surveys available in the WHO Household Energy database by region. 2

Modeling approaches investigated 2

Supplemental Material, Table S2. Percentage of population using solid fuels as main cooking fuel in 1990, 2000 and 2010, by country. 4

Supplemental Material, Table S3. Percentage of population using solid fuels as main cooking fuel in 1990, 2000 and 2010, by region. 9

Supplemental Material, Table S4. Population using solid fuels as main cooking fuel in 1990, 2000 and 2010, by region. 9

References 10

Supplemental Material, Table S1. Surveys available in the WHO Household Energy database by region.

Regions [a]	No. of countries (% region)/ territories	Population coverage	No. of surveys	% of surveys
Low and middle income countries				
Africa [b]	45 (98%)	100%	212	36
Americas	28 (97%)	100%	135	23
Eastern Mediterranean	15 (100%)	100%	38	6
Europe	20 (91%)	97%	48	8
Southeast Asia	11 (100%)	100%	54	9
Western Pacific	20 (95%)	100%	65	11
High income countries	13 (27%)	40%	25	4
Territories [c]	3	-	9	2
Total	**155**	**90%**	**586**	**100%**

Source: (WHO 2012a).

[a] Countries are grouped by WHO region and income category, for details see (WHO 2012b) p. 174-175. High income countries are defined as countries with a Gross National Income (GNI) of more than US$ 12,276.- per capita in 2010 according to the World Bank (The World Bank 2012) .

[b] Sub-Saharan

[c] Territories include Puerto Rico, Tokelau and Occupied Palestinian Territories.

Modeling approaches investigated

Based on previous experience (Mehta et al. 2006; WHO 2010), various linear regression approaches were explored. A wide range of developmental and energy related covariates thought to be related to solid fuel use were investigated. These included income, percentage of rural population, percentage of population with access to improved sanitation, and percentage of total energy consumption from fossil fuels. These models were not retained as they often did not closely follow empirical data.

Alternative modelling approaches considered but not retained included the family of Bayesian hierarchical/Gaussian process regression models (Finucane et al. 2011; Forouzanfar et al. 2011; Hogan et al. 2010; Rajaratnam et al. 2010b, 2010a). These models consist of several steps and

are generally used with covariates. They were not retained because they are complex, prone to subjectivity in selecting parameters, and because the model output is strongly influenced by regional trends where survey data are scarce, which may not allow for a ‘counter’ trend in certain countries.

As the outcome variable of interest is a percentage, we also explored multilevel models using the logit transformation of the outcome variable (Baum 2008). However, the modelled results often diverged from the empirical trends in the mid-range, and the logit model did not allow deviation from a monotonically decreasing or increasing trend over time. Therefore, a normal space model restricted to values between 0 and 0.98, and with smoothing applied around those values, was preferred.

Supplemental Material, Table S2. Percentage of population using solid fuels as main cooking fuel in 1990, 2000 and 2010, by country.

GBD Region [a]	Country	SFU(%) (95% confidence interval, %) 1990	2000	2010	Model[c]
Low and middle income countries [b]					
Africa [b]					
NA/ME	Algeria	14 (1, 28)	5 (0, 18)	0 (0, 11)	1
SSA Ce	Angola	98 (95, 100)	84 (71, 97)	55 (42, 68)	1
SSA We	Benin	98 (87, 100)	94 (81, 100)	91 (78, 100)	1
SSA So	Botswana	65 (52, 79)	50 (37, 63)	37 (25, 51)	1
SSA We	Burkina Faso	98 (94, 100)	98 (85, 100)	92 (79, 100)	1
SSA Ea	Burundi	98 (93, 100)	98 (87, 100)	96 (83, 100)	1
SSA We	Cameroon	94 (81, 100)	83 (70, 97)	75 (63, 89)	1
SSA We	Cape Verde	49 (36, 63)	39 (26, 52)	32 (19, 45)	1
SSA Ce	Central African Republic	98 (89, 100)	98 (85, 100)	96 (83, 100)	1
SSA We	Chad	98 (93, 100)	96 (83, 100)	88 (75, 100)	1
SSA Ea	Comoros	89 (76, 100)	79 (66, 92)	71 (58, 84)	1
SSA Ce	Congo	97 (84, 100)	86 (73, 99)	77 (64, 90)	1
SSA We	Côte d'Ivoire	87 (74, 100)	81 (69, 95)	78 (65, 91)	1
SSA Ce	DR of the Congo	98 (94, 100)	98 (86, 100)	93 (80, 100)	1
SSA Ce	Equatorial Guinea[d]	82 (78, 85)	79 (75, 82)	77 (74, 81)	2
SSA Ea	Eritrea	86 (73, 99)	72 (59, 85)	60 (47, 73)	1
SSA Ea	Ethiopia	93 (80, 100)	94 (81, 100)	98 (85, 100)	1
SSA Ce	Gabon	50 (37, 64)	36 (24, 50)	26 (13, 39)	1
SSA We	Gambia	98 (90, 100)	96 (83, 100)	91 (78, 100)	1
SSA We	Ghana	98 (88, 100)	91 (78, 100)	84 (71, 97)	1
SSA We	Guinea	98 (90, 100)	98 (86, 100)	97 (84, 100)	1
SSA We	Guinea-Bissau	98 (86, 100)	97 (84, 100)	98 (85, 100)	1
SSA Ea	Kenya	82 (69, 97)	80 (67, 93)	80 (68, 93)	1
SSA So	Lesotho	63 (50, 77)	61 (48, 74)	61 (48, 74)	1
SSA We	Liberia	98 (95, 100)	98 (89, 100)	98 (85, 100)	1
SSA Ea	Madagascar	98 (89, 100)	98 (86, 100)	98 (86, 100)	1
SSA Ea	Malawi	98 (89, 100)	98 (85, 100)	97 (84, 100)	1
SSA We	Mali	95 (82, 100)	96 (83, 100)	98 (87, 100)	1
SSA We	Mauritania	80 (67, 94)	68 (55, 81)	58 (45, 71)	1
As SE	Mauritius	19 (6, 33)	7 (0, 20)	0 (0, 11)	1
SSA Ea	Mozambique	98 (90, 100)	98 (85 , 100)	95 (82, 100)	1
SSA So	Namibia	74 (61, 89)	63 (50, 77)	55 (42, 68)	1
SSA We	Niger	98 (89, 100)	98 (85, 100)	96 (83, 100)	1
SSA We	Nigeria	74 (60, 88)	72 (60, 86)	74 (61, 87)	1
SSA Ea	Rwanda	98 (88, 100)	98 (86, 100)	98 (86, 100)	1
SSA We	Sao Tome and Principe	91 (78, 100)	80 (67, 93)	71 (58, 84)	1
SSA We	Senegal	81 (68, 95)	65 (52, 78)	51 (38, 64)	1
As SE	Seychelles	20 (7, 34)	7 (0, 20)	0 (0, 0, 10)	1
SSA We	Sierra Leone	93 (80, 100)	95 (82, 100)	98 (86, 100)	1
SSA So	South Africa	39 (26, 53)	25 (13, 39)	15 (2, 28)	1
SSA So	Swaziland	78 (65, 92)	65 (52, 79)	55 (43, 69)	1
SSA We	Togo	98 (94, 100)	98 (87, 100)	94 (81, 100)	1
SSA Ea	Uganda	98 (88, 100)	97 (84, 100)	96 (84, 100)	1
SSA Ea	Tanzania, United Republic	98 (86, 100)	96 (83, 100)	94 (81, 100)	1
SSA Ea	Zambia	95 (81, 100)	87 (74, 100)	83 (70, 96)	1

GBD Region [a]	Country	SFU(%) (95% confidence interval, %)			Model[c]
		1990	2000	2010	
SSA So	Zimbabwe	68 (55, 82)	66 (53, 79)	66 (53, 79)	1
Americas [b]					
LA So	Argentina	17 (4, 31)	6 (0, 20)	0 (0, 12)	1
Carib	Belize	29 (16, 44)	19 (7, 33)	12 (0, 25)	1
LA And	Bolivia, Plurinational States	45 (32, 59)	36 (23, 49)	29 (16, 42)	1
LA Tro	Brazil	19 (6, 33)	11 (0, 24)	6 (0, 19)	1
LA So	Chile	24 (10, 38)	14 (1, 27)	6 (0, 19)	1
LA Ce	Colombia	26 (13, 40)	19 (6, 32)	14 (1, 27)	1
LA Ce	Costa Rica	23 (10, 37)	13 (0, 26)	6 (0, 19)	1
Carib	Cuba	7 (0, 21)	6 (0 , 20)	9 (0, 22)	1
Carib	Dominica	42 (29, 56)	20 (7, 33)	1 (0, 14)	1
Carib	Dominican Republic	37 (23, 51)	20 (7, 34)	7 (0, 20)	1
LA And	Ecuador	27 (14, 41)	13 (1, 26)	2 (0, 15)	1
LA Ce	El Salvador	50 (37, 64)	35 (22, 48)	22 (9, 35)	1
Carib	Grenada	31 (17, 45)	11 (0, 25)	0 (0, 0)	1
LA Ce	Guatemala	64 (51, 78)	59 (46, 72)	57 (44, 70)	1
Carib	Guyana	26 (12, 40)	15 (2, 28)	7 (0, 20)	1
Carib	Haiti	98 (87, 100)	94 (81, 100)	91 (78, 100)	1
LA Ce	Honduras	68 (55, 82)	58 (45, 71)	51 (38, 64)	1
Carib	Jamaica	38 (25, 52)	23 (10, 36)	11 (0, 24)	1
LA Ce	Mexico	25 (12, 39)	18 (5, 31)	14 (1, 27)	1
LA Ce	Nicaragua	77 (63, 91)	64 (51, 77)	54 (41, 67)	1
LA Ce	Panama	25 (11, 39)	20 (7, 33)	18 (5, 31)	1
LA Tro	Paraguay	54 (41, 69)	50 (38, 54)	49 (36, 62)	1
LA And	Peru	62 (49, 76)	48 (35, 61)	36 (24, 50)	1
Carib	Saint Kitts and Nevis	27 (22, 33)	19 (13, 24)	14 (8, 19)	2
Carib	Saint Lucia	37 (24, 51)	14 (2, 28)	0 (0, 8)	1
Carib	Saint Vincent and Grenadines	69 (56, 83)	35 (22, 48)	3 (0, 16)	1
Carib	Suriname	30 (16, 44)	19 (6, 33)	12 (0, 25)	1
LA So	Uruguay	11 (0, 25)	4 (0, 17)	0 (0, 13)	1
LA Ce	Venezuela, Boliv. Rep.	15 (2, 29)	3 (0, 17)	0 (0, 8)	1
Eastern Mediterranean [b]					
NA/ME	Afghanistan	98 (87, 100)	91 (79, 100)	85 (72, 98)	1
SSA Ea	Djibouti	16 (2, 30)	13 (0, 26)	13 (0, 27)	1
NA/ME	Egypt	7 (0, 21)	1 (0, 15)	0 (0, 11)	1
NA/ME	Iran (Islamic Republic of)	12 (0, 26)	3 (0, 17)	0 (0, 11)	1
NA/ME	Iraq	11 (0, 26)	4 (0, 18)	0 (0, 13)	1
NA/ME	Jordan	12 (0, 26)	3 (0, 17)	0 (0, 11)	1
NA/ME	Lebanon	8 (0, 22)	0 (0, 12)	0 (0, 5)	1
NA/ME	Libyan Arab Jamahiriya	11 (0, 25)	1 (0, 14)	0 (0, 8)	1
NA/ME	Morocco	19 (6, 33)	9 (0, 23)	2 (0, 15)	1
As So	Pakistan	88 (74, 100)	74 (61, 88)	64 (51, 77)	1
SSA Ea	Somalia	98 (94, 100)	98 (87, 100)	95 (82, 100)	1
SSA Ea	Sudan	98 (94, 100)	93 (80, 100)	79 (66, 92)	1
NA/ME	Syrian Arab Republic	16 (3, 30)	5 (0, 18)	0 (0, 10)	1
NA/ME	Tunisia	18 (5, 32)	6 (0, 20)	0 (0, 11)	1
NA/ME	Yemen	48 (35, 62)	39 (26, 53)	33 (20, 46)	1
Europe [b]					
Eur Ce	Albania	64 (51, 78)	50 (37, 63)	39 (26, 52)	1

GBD Region [a]	Country	SFU(%) (95% confidence interval, %)			Model[c]
		1990	2000	2010	
As Ce	Armenia	85 (71, 97)	50 (37, 64)	19 (6, 32)	1
As Ce	Azerbaijan	52 (38, 66)	28 (15, 41)	7 (0, 20)	1
Eur Ea	Belarus	19 (6, 33)	8 (0, 21)	0 (0, 13)	1
Eur Ce	Bosnia and Herzegovina	58 (45, 72)	50 (37, 64)	45 (32, 58)	1
Eur Ce	Bulgaria	23 (17, 28)	13 (8, 19)	7 (2, 12)	2
As Ce	Georgia	55 (42, 69)	49 (36, 62)	46 (33, 59)	1
As Ce	Kazakhstan	29 (15, 43)	17 (4, 31)	9 (0, 22)	1
As Ce	Kyrgyzstan	51 (37, 65)	41 (28, 54)	34 (21, 47)	1
Eur Ea	Latvia	23 (10, 37)	13 (0, 26)	5 (0, 18)	1
Eur Ea	Lithuania	23 (17, 28)	13 (8, 19)	7 (2, 12)	2
Eur Ce	Montenegro	44 (31, 58)	35 (22, 48)	28 (15, 41)	1
Eur Ea	Republic of Moldova	28 (14, 42)	18 (5, 31)	11 (0, 24)	1
Eur Ce	Romania	35 (22, 49)	25 (12, 38)	17 (4, 30)	1
Eur Ea	Russian Federation	9 (0, 23)	3 (0, 17)	0 (0, 13)	1
Eur Ce	Serbia	51 (38, 65)	40 (27, 54)	32 (19, 45)	1
As Ce	Tajikistan	86 (73, 98)	59 (46, 72)	34 (22,48)	1
Eur Ce	TFYR of Macedonia	48 (34, 62)	39 (26, 52)	33 (20, 46)	1
NA/ME	Turkey	21 (7, 35)	10 (0, 24)	3 (0, 16)	1
As Ce	Turkmenistan	14 (1, 28)	3 (0, 16)	0 (0, 8)	1
Eur Ea	Ukraine	21 (8, 35)	10 (0, 24)	3 (0, 16)	1
As Ce	Uzbekistan	31 (18, 45)	20 (7, 33)	11 (0, 24)	1
South East Asia [b]					
As So	Bangladesh	91 (78, 100)	89 (76, 100)	91 (78, 100)	1
As So	Bhutan	78 (65, 93)	58 (45, 71)	40 (27, 53)	1
As Ea	DPR of Korea	98 (85, 100)	93 (80, 100)	91 (78, 100)	1
As So	India	87 (73, 99)	71 (58, 84)	58 (45, 71)	1
As SE	Indonesia	67 (53, 81)	59 (46, 73)	55 (42, 68)	1
As SE	Maldives	64 (15, 78)	35 (22, 48)	8 (0, 22)	1
As SE	Myanmar	98 (89, 100)	95 (83, 100)	92 (79, 100)	1
As So	Nepal	74 (61, 88)	77 (64, 90)	82 (69, 95)	1
As SE	Sri Lanka	89 (76, 100)	80 (68, 94)	75 (62, 88)	1
As SE	Thailand	63 (50, 77)	43 (30, 57)	26 (13, 39)	1
As SE	Timor-Leste	96 (82, 100)	92 (79 , 100)	92 (79, 100)	1
Western Pacific [b]					
As SE	Cambodia	98 (88, 100)	94 (81, 100)	89 (76, 100)	1
As Ea	China	64 (51, 78)	53 (41, 67)	46 (33, 59)	1
Ocea	Cook Islands	23 (9, 37)	12 (0, 25)	4 (0, 17)	1
Ocea	Fiji	55 (42, 69)	44 (32, 58)	37 (24, 50)	1
Ocea	Kiribati	66 (54, 77)	55 (43, 66)	46 (35, 57)	2
As SE	Lao PDR	96 (83, 100)	95 (82, 100)	96 (83, 100)	1
As SE	Malaysia	22 (9, 36)	8 (0, 21)	0 (0, 10)	1
Ocea	Marshall Islands	20 (6, 34)	24 (11, 38)	32 (19, 45)	1
Ocea	Micronesia (Fed. St. of)	55 (42, 70)	47 (34, 60)	41 (28, 54)	1
As Ce	Mongolia	81 (68, 95)	75 (62, 89)	72 (60, 86)	1
Ocea	Nauru	5 (0, 19)	3 (0, 17)	5 (0, 18)	1
Western Pacific [b]					
Ocea	Niue	46 (32, 60)	23 (10, 37)	4 (0, 17)	1
Ocea	Palau	10 (0, 24)	1 (0, 15)	0 (0, 8)	1
Ocea	Papua New Guinea	95 (82, 100)	83 (70, 96)	73 (60, 86)	1
As SE	Philippines	60 (47, 74)	53 (41, 67)	50 (37, 63)	1

GBD Region [a]	Country	SFU(%) (95% confidence interval, %)			Model[c]
		1990	2000	2010	
Ocea	Samoa	70 (57 , 84)	60 (47, 74)	53 (40, 66)	1
Ocea	Solomon Islands	90 (77, 100)	88 (76, 100)	90 (77, 100)	1
Ocea	Tonga	72 (58, 86)	56 (43, 69)	43 (30, 56)	1
Ocea	Tuvalu	67 (54, 81)	42 (29, 55)	19 (6, 32)	1
Ocea	Vanuatu	83 (69, 97)	82 (69, 95)	84 (71, 97)	1
As SE	Viet Nam	97 (85, 100)	76 (63, 89)	56 (43, 69)	1
High income countries [b]					
Eur We	Andorra	<5	<5	<5	3
Carib	Antigua and Barbuda	14 (1, 28)	5 (0, 18)	0 (0, 12)	1
AusAs	Australia	<5	<5	<5	3
Eur We	Austria	<5	<5	<5	3
Carib	Bahamas	<5	<5	<5	3
NA/ME	Bahrain	<5	<5	<5	3
Carib	Barbados	3 (0, 17)	0 (0, 13)	0 (0, 11)	1
Eur We	Belgium	<5	<5	<5	3
AP HI	Brunei Darussalam	<5	<5	<5	3
NA HI	Canada	<5	<5	<5	3
Eur Ce	Croatia	27 (14, 41)	16 (3, 29)	8 (0, 21)	1
Eur We	Cyprus	<5	<5	<5	3
Eur Ce	Czech Republic	18 (5, 32)	6 (0, 20)	0 (0, 10)	1
Eur We	Denmark	<5	<5	<5	3
Eur Ea	Estonia	28 (15, 42)	18 (5, 32)	11 (0, 24)	1
Eur We	Finland	<5	<5	<5	3
Eur We	France	<5	<5	<5	3
Eur We	Germany	<5	<5	<5	3
Eur We	Greece	<5	<5	<5	3
Eur Ce	Hungary[e]	2 (1, 2)	0 (0, 1)	<5	2, 3
Eur We	Iceland	<5	<5	<5	3
Eur We	Ireland	<5	<5	<5	3
Eur We	Israel	<5	<5	<5	3
Eur We	Italy	<5	<5	<5	3
AP HI	Japan	<5	<5	<5	3
NA/ME	Kuwait	<5	<5	<5	3
Eur We	Luxembourg	<5	<5	<5	3
Eur We	Malta	<5	<5	<5	3
Eur We	Monaco	<5	<5	<5	3
Eur We	Netherlands	<5	<5	<5	3
AusAs	New Zealand	<5	<5	<5	3
Eur We	Norway	<5	<5	<5	3
NA/ME	Oman	<5	<5	<5	3
Eur Ce	Poland[e]	2 (1, 2)	0 (0, 1)	<5	2, 3
Eur We	Portugal	<5	<5	<5	3
NA/ME	Qatar	8 (0, 22)	2 (0, 15)	0 (0, 12)	1
High income countries [b]					
AP HI	Republic of Korea	20 (7, 24)	0 (0, 5)	0 (0, 5)	1
Eur We	San Marino	<5	<5	<5	3
NA/ME	Saudi Arabia	<5	<5	<5	3
AP HI	Singapore	<5	<5	<5	3
Eur Ce	Slovakia	19 (6, 33)	7 (0, 21)	0 (0, 11)	1
Eur Ce	Slovenia	24 (10, 38)	12 (0, 26)	4 (0, 17)	1

GBD Region [a]	Country	SFU(%) (95% confidence interval, %)			Model[c]
		1990	2000	2010	
Eur We	Spain[f]	<5	<5	<5	3
Eur We	Sweden	<5	<5	<5	3
Eur We	Switzerland	<5	<5	<5	3
Carib	Trinidad and Tobago	19 (6, 33)	7 (0, 20)	0 (0, 11)	1
NA/ME	United Arab Emirates	14 (1, 28)	3 (0, 17)	0 (0, 8)	1
Eur We	United Kingdom	<5	<5	<5	3
NA HI	United States of America[f]	<5	<5	<5	3

SFU: solid fuel use. DPR: Democratic People's Republic. DR: Democratic Republic. PDR: People's Democratic Republic. Boliv. Rep.: Bolivarian Republic. TFYR: The former Yugoslav Republic. Fed. St.: Federated States. AP HI: Asia Pacific, High Income; As Ce: Asia Central; As So: Asia Southern; Asia SE: Asia Southeast; AusAs: Australasia; Carib: Caribbean; ; Eur Ce: Europe Central; Eur Ea: Europe East; Eur We; Europe West; LA And: Latin America, Andean; LA Cen: Latin America Central; LA So: Latin America, Southern; LA Trop: Latin America, Tropical; NA HI: North America High Income; NA/ME: North Africa/ Middle East; Ocea: Oceania; SSA CE: Sub-Saharan Africa, Central; SSA Ea: Sub-Saharan Africa, East; SSA So: Sub-Saharan Africa, Southern; SSA We: Sub-Saharan Africa, West.

[a] 21 Global Burden of Disease regions (Institute for Health Metrics and Evaluation et al. 2009).

[b] WHO regions and income category [see (WHO 2012b) p. 174-175]. Africa refers to Sub-Saharan Africa.

[c] Type of model used: 1 multilevel model; 2 regional estimates: these estimates provide indicative values only and are not based on the data of the specific country; 3 Countries with no survey data classified as high income countries, for which assumption was made that solid fuel use for cooking is <5%. Note that this does not strictly mean that no solid fuels are used, but if they are, they are used in stoves or devices that do not result in exposure.

[d] Equatorial Guinea is included in Africa and not grouped with high income countries for the purpose of this analysis.

[e] Hungary and Poland qualify as high-income countries in 2010 according to the World Bank (The World Bank 2012).

[f] Survey data for Spain and United States of America was available and was included in the model, yet these two countries are reported as high-income with no data and SFU <5% (consistent with model output).

Supplemental Material, Table S3. Percentage of population using solid fuels as main cooking fuel in 1990, 2000 and 2010, by region.

Region [a]	SFU (%) (95% confidence interval, %)		
	1990	2000	2010
Low and middle income countries			
Africa [b]	82 (78,85)	79 (75, 82)	77 (74, 81)
Americas	27 (22, 33)	19 (13, 24)	14 (8, 19)
Eastern Mediterranean	48 (43,54)	40 (35, 46)	35 (29, 40)
Europe	23 (17, 28)	13 (8, 19)	7 (2, 12)
South East Asia	83 (74, 93)	71 (62, 80)	61 (52, 70)
Western Pacific	66 (54, 77)	55 (43, 66)	46 (35, 57)
High income countries	2 (1, 2)	0 (0， 1)	0 (0， 1)
**World**	**53 (49 , 56)**	**46 (42 , 49)**	**41 (37 , 44)**

SFU: solid fuel use.

[a] Countries are grouped by WHO region and income category [see (WHO 2012b) p. 174-175].

[b] Sub-Saharan Africa

Supplemental Material, Table S4. Population using solid fuels as main cooking fuel in 1990, 2000 and 2010, by region.

Region [a]	Population exposed [millions] (95% confidence intervals)		
	1990	2000	2010
Low and middle income countries			
Africa [b]	413 (395, 431)	517 (494, 539)	646 (617, 675)
Americas	119 (95, 143)	97 (69, 124)	80 (49, 111)
Eastern Mediterranean	171 (152, 190)	182 (159, 206)	190 (162, 219)
Europe	89 (66, 112)	53 (31, 75)	28 (7, 50)
South East Asia	1100 (979, 1221)	1112 (972, 1253)	1097 (934, 1260)
Western Pacific	865 (711, 1020)	809 (643, 975)	739 (563, 914)
High income countries	15 (10, 21)	3 (0, 9)	1 (0, 7)
**World**	**2772 (2571, 2973)**	**2773 (2551, 2996)**	**2780 (2534, 3027)**

[a] Countries are grouped by WHO region and income category [see (WHO 2012b) p. 174-175].

[b] Sub-Saharan Africa

References

Baum C. 2008. Stata tip 63: Modeling proportions. The Stata Journal 8:299–303.

Finucane MM, Stevens GA, Cowan MJ, Danaei G, Lin JK, Paciorek CJ, et al. 2011. National, regional, and global trends in body-mass index since 1980: systematic analysis of health examination surveys and epidemiological studies with 960 country-years and 9·1 million participants.Lancet 377:557–567; doi:10.1016/S0140-6736(10)62037-5.

Forouzanfar MH, Foreman KJ, Delossantos AM, Lozano R, Lopez AD, Murray CJL, et al. 2011. Breast and cervical cancer in 187 countries between 1980 and 2010: a systematic analysis. Lancet 378:1461–1484; doi:10.1016/S0140-6736(11)61351-2.

Hogan MC, Foreman KJ, Naghavi M, Ahn SY, Wang M, Makela SM, et al. 2010. Maternal mortality for 181 countries, 1980–2008: a systematic analysis of progress towards Millennium Development Goal 5. Lancet 375:1609–1623; doi:10.1016/S0140-6736(10)60518-1.

Institute for Health Metrics and Evaluation, Harvard University, Johns Hopkins University, University of Queensland, WHO. 2009. Operations Manual - Final Draft. Available : http://www.gbd.unsw.edu.au/gbdweb.nsf/resources/OperationsManual/$file/final+GBD+operations+manual.pdf [accessed 23 March 2012]

Mehta S, Gore F, Prüss-Üstün A, Rehfuess E, Smith K. 2006. Modeling household solid fuel use towards reporting of the Millennium Development Goal indicator. Energy for Sustainable Development 10:36–45; doi:10.1016/S0973-0826(08)60542-6.

Rajaratnam JK, Marcus JR, Flaxman AD, Wang H, Levin-Rector A, Dwyer L, et al. 2010a. Neonatal, postneonatal, childhood, and under-5 mortality for 187 countries, 1970–2010: a systematic analysis of progress towards Millennium Development Goal 4. The Lancet 375:1988–2008; doi:10.1016/S0140-6736(10)60703-9.

Rajaratnam JK, Marcus JR, Levin-Rector A, Chalupka AN, Wang H, Dwyer L, et al. 2010b. Worldwide mortality in men and women aged 15–59 years from 1970 to 2010: a systematic analysis. Lancet 375:1704–1720; doi:10.1016/S0140-6736(10)60517-X.

The World Bank. 2012. Data - How we classify countries. Available: http://data.worldbank.org/about/country-classifications [accessed 23 March 2012].

WHO (World Health Organization). 2010. World health statistics 2010. Available: http://www.who.int/gho/publications/world_health_statistics/2010/en/index.html [accessed 14 March 2013].
WHO (World Health Organization). 2012a. Global Health Observatory. WHO. Available: http://www.who.int/gho/en/ [accessed 23 March 2012].
WHO (World Health Organization). 2012b. World health statistics 2012. Available: http://www.who.int/gho/publications/world_health_statistics/2012/en/index.html [accessed 14 March 2013].
